# Gastrocolic fistula secondary to primary gastric lymphoma

**DOI:** 10.11604/pamj.2014.17.15.3787

**Published:** 2014-01-15

**Authors:** Toufik Berri

**Affiliations:** 1 Department of Surgery, Tourabi Boudjemaa Hospital, Bechar, Algeria

**Keywords:** Gastrocolic fistula, gastric lymphoma, stomach

## Image in medicine

Gastrocolic fistula (GCF) is an abnormal communication between a portion of the stomach and the transverse colon. It is a rare entity and could be due to benign or malignant disease. The gastric or colonic adenocarcinoma is the most common malignant cause, while lymphoma is rarely reported. The cornerstone for detecting the fistula remains the barium enema. Barium meal and computed tomography are alternatives for the diagnosis. Gastroscopy and colonoscopy are not first-line examinations to bring out the GCF, but they must be used to obtain cytological and biopsy materials. The therapy for GCF remains surgical. A 36-year-old woman had a non-Hodgkin's gastric lymphoma for 15 years ago treated by chemotherapy. After complete remission, she presented with abdominal pain, fecal halitosis, feculent vomiting, chronic diarrhea and weight loss of 15 kg. A palpable mass in the left hypochondriac region was found on abdominal examination. Gastroscopy revealed a vegetating tumor on the fundus with fistulous orifice in the great curvature of the stomach. Barium meal showed early opacification of the colon and a fistula between the stomach and transverse colon (A). Abdominal CT demonstrated a wall thickening of the great curvature of the stomach with GCF (B). There is neither adjacent nor distant involvement by the lymphoma. According to Lugano staging system, the lymphoma was classified as stage IIE. The patient was prepared for an *en bloc* resection of the involved stomach and colon but she died before undergoing the operation.

**Figure 1:**
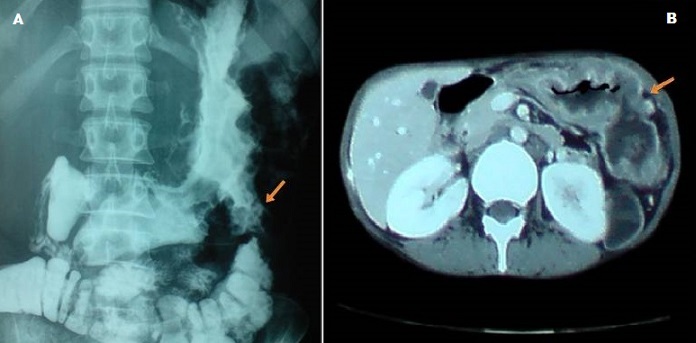
A) Barium meal; B) Abdominal computed tomography. The arrow shows the gastrocolic fistula

